# A nomogram model for identifying cognitive frailty in elderly patients with chronic diseases: a multicenter cross-sectional study

**DOI:** 10.3389/fpubh.2026.1815512

**Published:** 2026-07-28

**Authors:** Shouqiang Huang, Huan Liu, Qingwei Liu, Lan Luo, Wuxian Chen, Zhiqing Zhou, Xinyu Hu, Xu Qin, Caixia Xing, Xiubin Tao, Lingling Pan, Ming Zhang

**Affiliations:** 1 Department of Ophthalmology, Wuhu Second People's Hospital, Wuhu, Anhui, China; 2 Department of Hemodialysis, The First Affiliated Yijishan Hospital of Wannan Medical College (Yijishan Hospital of Wannan Medical College), Wuhu, Anhui, China; 3 Department of Nursing, Shandong Provincial Hospital Affiliated to Shandong First Medical University (Shandong Provincial Hospital), Jinan, Shandong, China; 4 Department of Cardiology, The First Affiliated Hospital of Guangxi Medical University, Nanning, Guangxi, China; 5 Department of Nursing, The First Affiliated Yijishan Hospital of Wannan Medical College (Yijishan Hospital of Wannan Medical College), Wuhu, Anhui, China; 6 Department of Cardiovascular, The Second Affiliated Hospital of Wangnan Medical College, Wuhu, Anhui, China; 7 Department of Interventional, The First Affiliated Hospital of Wannan Medical College (Yijishan Hospital of Wannan Medical College), Wuhu, Anhui, China; 8 School of Nursing, Wanna Medical College, Wuhu, Anhui, China; 9 Department of Cardiology, The First Affiliated Yijishan Hospital of Wannan Medical College (Yijishan Hospital of Wannan Medical College), Wuhu, Anhui, China; 10 School of Innovation and Entrepreneurship, Wanna Medical College, Wuhu, Anhui, China; 11 Key Laboratory of Philosophy and Social Science of Anhui Province on Adolescent Mental Health and Crisis Intelligence Intervention, Hefei Normal University, Hefei, China

**Keywords:** cognitive frailty, elderly patients with chronic diseases, nomogram, prediction, risk factors

## Abstract

**Objectives:**

In the context of population aging, the prevalence of cognitive frailty among elderly individuals with chronic diseases is steadily increasing. Cognitive frailty is a prevalent geriatric condition that significantly impacts the health of elderly patients with chronic diseases. This study aimed to explore the influencing factors of cognitive frailty in elderly patients with chronic diseases and to development and Internal Validation of a Nomogram for Screening Prevalent Cognitive Frailty.

**Methods:**

From March 2025 to August 2025, a total of 752 elderly patients with chronic diseases (age ≥ 60 years) were selected from the First Affiliated Hospital of Wannan Medical College, the Second People’s Hospital of Wuhu, and the First Affiliated Hospital of Guangxi Medical University. The assessment utilized the Overall Cognition Ascertain Dementia-8, FRAIL Scale, Clinical Physiological Resilience, Oral Frailty Scale, SARC-F Questionnaire, and PHQ-2 Depression Scale. The logistic regression model was applied to explore the associated factors of cognitive frailty in elderly patients with chronic diseases. The model’s performance was evaluated using various measures, including the area under the receiver operating characteristic curve, the calibration curve, the Hosmer–Lemeshow test, and decision curve analysis.

**Results:**

A total of 752 elderly patients with chronic diseases were included, among whom 171 (22.7%) had cognitive frailty. Gender, age, oral frailty, sarcopenia, depressive status, and clinical physiological resilience were identified as influencing factors of cognitive frailty in elderly patients with chronic diseases (*p* < 0.05). In the training set, the AUC was 0.856 (95% CI: 0.823–0.890), while in the validation set, the AUC was 0.886 (95% CI: 0.840–0.932). Additionally, the test results showed that the model exhibited good fit in both the training set (*χ*^2^ = 10.154, *p =* 0.2054) and the validation set (*χ*^2^ = 5.156, *p =* 0.741). The Brier scores for the training set and the validation set were 0.128 and 0.104, respectively.

**Conclusion:**

Elderly patients with chronic diseases have a higher incidence of cognitive frailty. The nomogram model in this multicenter cross-sectional study may help identify hospitalized older patients with chronic diseases who are likely to have cognitive frailty and may require further assessment.

## 1 Introduction

On July 24, 2025, the Ministry of Civil Affairs of the People’s Republic of China and the National Working Commission on Aging released the “2024 National Development Report on Aging.” The data showed that by the end of 2024, the population aged 60 and above in China had reached 310.31 million, accounting for 22.0% of the total population; the population aged 65 and above was 220.23 million, accounting for 15.6% of the total population (1). By 2034, the proportion of the population aged 60 and above will exceed 30%, and by 2055, it will surpass 40%, marking China’s entry into a super-aged society (2). With the further acceleration of China’s population aging process, a series of serious health issues among elderly patients with chronic diseases have continuously emerged, such as cognitive frailty (3). Cognitive frailty is associated with adverse health outcomes such as falls and mortality (4). This not only imposes a heavy economic burden on individuals, families, and healthcare institutions, but also has a comprehensive, profound, and extensive impact on the overall socio-economic development (5). Research has shown that cognitive frailty could potentially be reversed (6). On May 26, 2023, the General Office of the National Health Commission of the People’s Republic of China issued the “Notice on Launching the Dementia Prevention and Promotion Action (2023–2025),” which clearly states that early screening, early detection, and early intervention of cognitive function in the elderly are crucial measures to reduce or delay the onset of dementia (7). Therefore, conducting research on the current status of cognitive frailty in elderly patients with chronic diseases and the factors influencing cognitive frailty, as well as implementing early screening and targeted intervention methods, is crucial for preventing or delaying adverse health outcomes associated with cognitive frailty (8).

In 2013, an international consensus panel composed of the International Alzheimer’s Association/International Association of Gerontology and Geriatrics (I.A.N.A./I.A.G.G.) proposed the concept of cognitive frailty (CF), defining it as a combined manifestation of cognitive impairment and physical frailty in non-demented elderly individuals (9). A systematic review and meta-analysis published in the International Journal of Nursing Studies by Yiming Qiu and colleagues in 2022 revealed that the pooled prevalence of cognitive frailty syndrome among community-dwelling older adults was 9%. Subgroup analysis results indicated that the pooled prevalence of cognitive frailty was 6% (95% CI: 4–8%) between 2012 and 2017, and 11% (95% CI: 9–14%) between 2018 and 2020 (10). It is well known that the prevalence of cognitive frailty is significantly higher among elderly patients with chronic diseases compared to healthy elderly populations, and this phenomenon is commonly observed across various chronic conditions. For instance, chronic diseases such as cardiovascular diseases, type 2 diabetes, and hypertension not only increase the risk of cognitive decline in patients but may also accelerate the progression of cognitive frailty (11, 12). CF is linked to an increased likelihood of hospitalization, disability, and mortality (13, 14). Furthermore, research indicates that CF can serve as an indicator of general dementia, which encompasses vascular dementia (15). A nationwide cross-sectional study involving 12 provinces and municipalities indicates that in China, there are 15.07 million people aged 60 and above suffering from dementia, with an additional 38.77 million experiencing mild cognitive impairment (16). Cognitive impairment not only affects families but may also impose a significant socioeconomic burden on society. Taking Alzheimer’s disease patients as an example, the annual socioeconomic cost per patient is as high as $19,144.36 (17). Previous studies have shown that cognitive frailty in the elderly not only increases the risk of adverse health outcomes such as functional impairment, disability rates, hospitalization, and all-cause mortality (18), but also significantly impacts the increased demand for long-term care (19), shortens healthy life expectancy (20), and exacerbates the medical burden on society and families (21, 22).

The development of cognitive frailty in the elderly is influenced by a combination of various factors, encompassing demographic characteristics, lifestyle, diseases, biochemical indicators, and social support. Previous studies have explored the impact of certain lifestyle factors on cognitive impairment, including smoking (23), excessive alcohol consumption (24), lack of physical exercise (25), and unhealthy dietary habits (25, 26). With the acceleration of population aging, the prevalence of diseases associated with aging in elderly patients with chronic conditions, such as frailty and cognitive decline, has significantly increased (27). Unfortunately, to the best of our research group’s knowledge, few studies have specifically focused on exploring the influencing factors of cognitive frailty in elderly hospitalized patients with chronic diseases. Therefore, investigating and exploring the current status of cognitive frailty and its influencing factors among elderly inpatients with chronic diseases helps to fill the research gap in this field, enrich and improve the theoretical system of health management for elderly inpatients with chronic diseases, and provide a theoretical basis for targeted interventions and improvement of cognitive frailty in this patient population.

Physical resilience refers to an individual’s ability to recover from challenges such as illness, injury, or age-related decline (28) and plays a crucial role in promoting the health of older adults. In 2015, the National Institute on Aging discussed and proposed that the level of physiological resilience is crucial for the clinical outcomes of elderly individuals after exposure to stressors such as infections, trauma, etc. (29). Physical resilience emphasizes the positive attributes that promote health in older adults (30). Higher levels of physical resilience lead to positive clinical outcomes, such as rapid recovery after surgery. Conversely, poor physical resilience may result in adverse clinical outcomes, such as prolonged course of illness/hospitalization, or even death (31). The decline in physical resilience is also a manifestation of premature aging and a consequence of the failure of the homeostatic network in the stress response system (30, 32, 33). For older adults who are at risk of impaired physical function, physical resilience is a crucial component in maintaining their health (34, 35). Understanding the relationship between clinical physiological resilience and cognitive frailty helps identify individuals at higher risk of cognitive frailty and facilitates the development of targeted interventions for the prevention and effective management of cognitive frailty. Therefore, it is necessary to explore the relationship between clinical physiological resilience and cognitive frailty in elderly patients with chronic diseases, in order to enrich research in this field and provide a foundation for future related studies.

Oral frailty refers to a condition in the elderly where the decline in oral function leads to the accumulation of adverse oral conditions to a certain extent. It is a slowly occurring process related to aging, encompassing various oral conditions such as tooth loss, poor oral hygiene, and reduced oral function. This condition is often accompanied by eating dysfunction, decreased attention to oral health, and a decline in both physical and mental functions (36). Oral frailty is an early manifestation of geriatric frailty (37), including a reduction in the number of teeth (i.e., tooth loss) and a decrease in attention to oral health. Research has found that for each tooth lost, the rate of brain degeneration in elderly individuals accelerates by 1 year (38). Additionally, a meta-analysis revealed that subjects with fewer than 20 functional teeth have a 26% increased risk of cognitive decline and a 22% higher risk of dementia (39). Furthermore, poor oral health is associated with an increased ability of oral microbiota to reach the brain, thereby affecting cognitive function and increasing the risk of dementia in the elderly (40). In recent years, the correlation between oral frailty and cognitive dysfunction has become a global research hotspot (41, 42). However, in China, attention to these two aspects is still in its infancy. Therefore, the significance of oral frailty has garnered unprecedented attention from clinicians and researchers.

Sarcopenia was first proposed in 1989 by American professor Irwin Rosenberg (43). In recent years, sarcopenia, as a significant health issue among the elderly population, has gradually become a focal point in preventive medicine and geriatric research. Sarcopenia is a geriatric syndrome characterized by progressive and generalized loss of skeletal muscle mass, strength, and function, and has been officially recognized as a disease by the World Health Organization (WHO) (44). Sarcopenia and cognitive frailty share some common risk factors, such as comorbid chronic diseases in the elderly and elderly diabetes, both of which can adversely affect sarcopenia and cognitive frailty. Active prevention and treatment of sarcopenia will bring significant benefits to the elderly, their families, and society, especially interventions in the early stages or pre-sarcopenia stage (45). A meta-analysis found that sarcopenia is more likely to be associated with cognitive impairment (46). Obesity, oxidative stress, and cerebrovascular diseases are predisposing factors for sarcopenia and may also be common risk factors for cognitive impairment (47).

Depressive symptoms are one of the common illnesses among older adults, which not only negatively impact their quality of life but are also associated with an increased risk of mortality (48, 49). A survey found that the prevalence of depressive symptoms among elderly Chinese reached as high as 44.5% (50). Studies have shown that cognitive frailty is significantly associated with depressive symptoms (51). The interaction between depressive symptoms and chronic diseases exacerbates the risk of cognitive frailty in the elderly. Depressive symptoms activate the hypothalamic–pituitary–adrenal (HPA) axis and promote the release of pro-inflammatory cytokines, triggering neuroinflammation and neuronal damage, which in turn impair brain regions closely associated with cognitive function, such as the hippocampus and prefrontal cortex (52). With the accelerating pace of global aging, the various health issues of elderly patients with chronic diseases have become increasingly prominent, among which the association between cognitive frailty and depression symptoms is particularly noteworthy. As one of the most common psychological disorders among elderly chronic disease patients, depression symptoms not only significantly reduce the quality of life of patients but may also accelerate the decline of their cognitive functions, thereby exacerbating the degree of cognitive frailty.

In light of the aforementioned research, it is necessary to conduct cognitive frailty screening and identify its associated risk factors in elderly patients with chronic diseases. A deeper understanding of the related factors of cognitive frailty in hospitalized elderly patients with chronic diseases will aid in developing targeted interventions to prevent adverse outcomes, provide a scientific reference for establishing a cognitive frailty warning model, and potentially delay and reverse the state of cognitive frailty in elderly patients with chronic diseases. A nomogram is a quantitative analytical graph that represents the functional relationships among multiple variables using a cluster of non-intersecting line segments in a plane coordinate system. As a practical and user-friendly predictive tool, it is widely popular for its advantage of allowing direct graphical estimation of a variable’s value, such as a patient’s indicator score or survival probability. By integrating the specific impacts of various influencing factors on the outcome variable through a multivariate regression model, the nomogram assigns corresponding scores to different value levels of each influencing factor, thereby enabling rapid scoring of each risk factor and convenient summarization of the total score (53). Therefore, this study aims to utilize multicenter data with sufficient sample sizes and easily accessible psychological, physiological, and general demographic data to analyze the risk factors associated with cognitive frailty in elderly patients with chronic diseases. Furthermore, the research seeks to develop a nomogram model for predicting the risk of cognitive frailty in elderly chronic disease patients during hospitalization. This study is intended to provide reference and valuable guidance for clinicians in identifying high-risk patients for cognitive frailty, enabling targeted multidisciplinary interventions.

## 2 Materials and methods

The objective of this study is to develop and internally validate a nomogram for screening prevalent cognitive frailty in elderly patients with chronic diseases. This study employed a descriptive cross-sectional research design. This design was chosen because it enables the simultaneous screening of multiple variables associated with cognitive frailty in elderly patients with chronic diseases. By collecting data at a single time point, it conveniently and rapidly provides an exploration of the associations between different factors and cognitive frailty outcomes.

**Figure 1:**
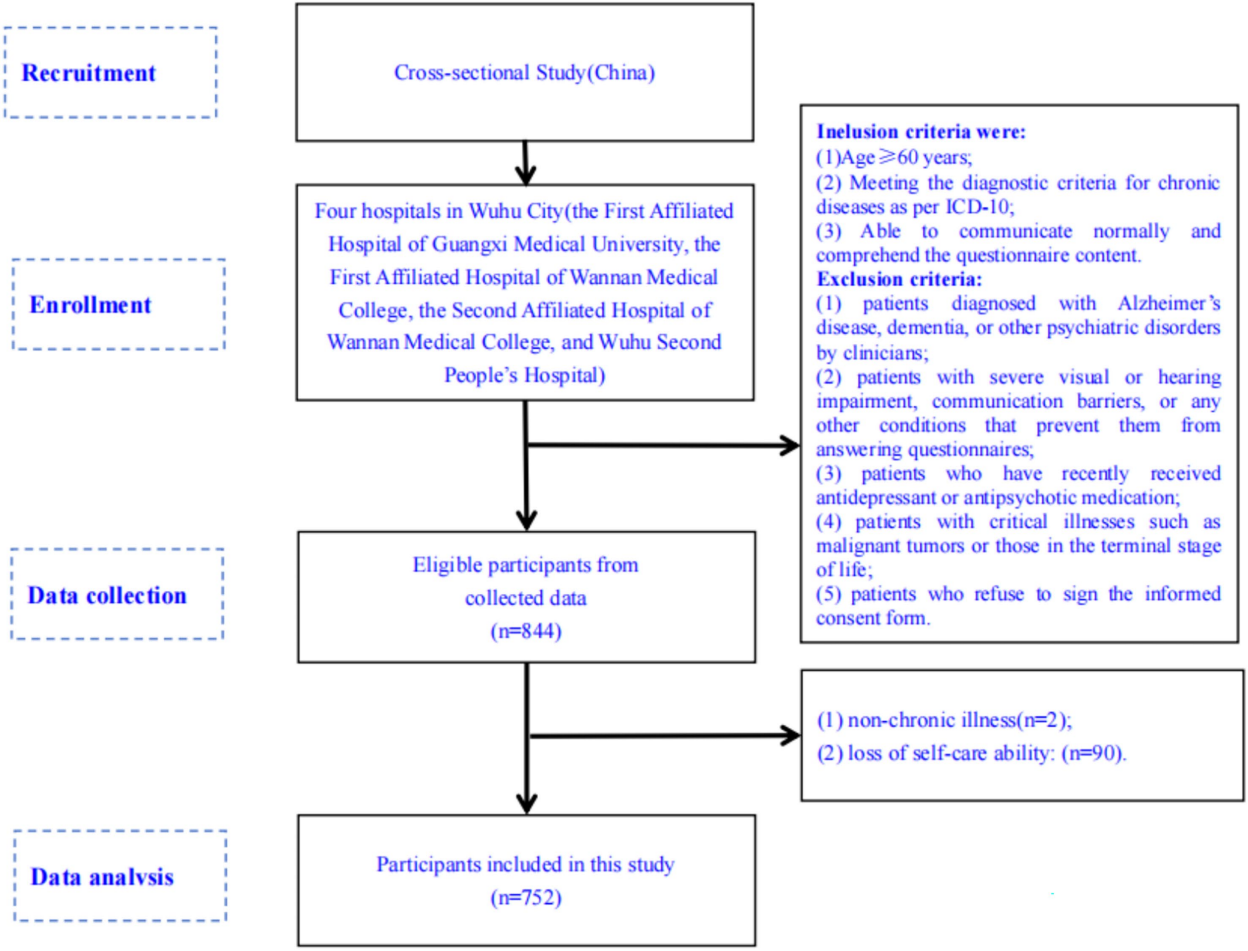
Sample selection process for this cross-sectional study.

This study adopted a descriptive cross-sectional research design, strictly adhering to the Strengthening the Reporting of Observational Studies in Epidemiology (STROBE) checklist to guide the data collection, analysis, and interpretation of findings in this cross-sectional study. This cross-sectional study conducted a questionnaire survey among 752 elderly participants with chronic diseases. The study was carried out from March 29, 2025, to August 2, 2025, and all participants were selected from the First Affiliated Hospital of Guangxi Medical University, the First Affiliated Hospital (Yijishan Hospital) of Wannan Medical College, the Second Affiliated Hospital of Wannan Medical College, and the Second People’s Hospital of Wuhu City. All participants were elderly inpatients with chronic diseases, and a total of 752 individuals were ultimately included in the study. Inclusion criteria: (1) Age ≥60 years; (2) Meeting the diagnostic criteria for chronic diseases as per ICD-10 (International Classification of Diseases, Tenth Revision); (3) Able to communicate normally and comprehend the questionnaire content. The exclusion criteria include: (1) patients diagnosed with Alzheimer’s disease, dementia, or other psychiatric disorders by clinicians; (2) patients with severe visual or hearing impairment, communication barriers, or any other conditions that prevent them from answering questionnaires; (3) patients who have recently received antidepressant or antipsychotic medication; (4) patients with critical illnesses such as malignant tumors or those in the terminal stage of life; and (5) patients who refuse to sign the informed consent form (Figure 1).

This study adopted a cross-sectional research design, with the sample size calculated based on the principle of at least 10 events per variable (EPV). Given that the study encompasses 11 variables and an anticipated loss to follow-up rate ranging between 10 and 20%, a minimum of 110 samples was required for model construction. Since the modeling sample size accounted for 70% of the total sample in this cross-sectional study, the total sample size should be at least 330. Ultimately, we included 752 samples, which adequately met the aforementioned requirements.

### 2.1 Measurement

#### 2.1.1 Demographic characteristics questionnaire

In this study, the demographic characteristics questionnaire was independently designed by the researchers based on the research objectives, following a comprehensive review of both domestic and international literature and expert consultations. It primarily covers variables such as gender, place of residence, age, duration of illness, marital status, educational level, years of work experience, number of hospitalizations in the past 2 years, and self-rated health.

#### 2.1.2 Cognitive frailty

The cognitive frailty of older chronically ill patients was assessed using the Overall Cognition Assessment Dementia-8 (AD8). The AD8 is a concise and readily administered screening tool. We used the validated Chinese version of the AD8, whose psychometric properties have been well established (54). The AD8 scale comprises eight questions that assess judgment, interests, engagement in activities, frequency of repetitive conversations, learning capacity, memory for dates and appointments, financial management skills, and daily cognitive processes. A total of ≥2 points is considered indicative of cognitive impairment (55).

#### 2.1.3 Frail

The frailty status of older chronically ill patients was assessed using the FRAIL scale, which consists of five questions: (1) Fatigue: Have you felt tired most of the time in the past month? (2) Low endurance: Is it difficult to climb a flight of stairs without help? Walking: Is it difficult to walk 100 meters without help? Illness: Do you have five or more diseases? Weight Loss: Have you lost at least 5% of your body weight in the past year? Each item was scored on a scale of 0–1, with a score of 1 for “yes” and a score of 0 for “no” (56). The maximum score on the scale is 5, and a test result of ≥3 indicates a higher risk of frailty.

#### 2.1.4 Clinical physiological resilience

Clinical physiological resilience is assessed using the CHEES scale, which was developed in 2024 by scholars, including Li Jing from the National Clinical Research Center for Geriatric Diseases, Department of Geriatrics, Xuanwu Hospital, Capital Medical University (57). The CHEES scale encompasses three dimensions: intrinsic personal capacity, adaptability to change, and external support, comprising a total of 14 items. Each item is scored using a Likert 5-point scale, ranging from “strongly disagree” to “strongly agree,” with scores from 1 to 5 (1 = strongly disagree, 2 = disagree, 3 = uncertain, 4 = agree, 5 = strongly agree). Item 4 is reverse-scored. The total score of the CHEES scale ranges from 14 to 70, with higher scores indicating better physiological resilience. Previous evidence has shown that the Chinese version of the CHEES scale demonstrates reliability and validity among elderly inpatients in China.

#### 2.1.5 Oral frailty

In this cross-sectional study, the Oral Frailty Index (OFI-8) was used to assess the participants’ level of oral frailty. The OFI-8 was developed by the Japanese Dental Association (58), and the Chinese version was translated by Chen Zongmei et al. in 2023 (59). The Cronbach’s alpha coefficient for the Chinese version of the OFI-8 was 0.949. The OF1-8 scale consists of five dimensions: masticatory ability (2 items), swallowing function (2 items), denture use (1 item), social participation (1 item), and oral health-related behaviors (2 items), totaling 8 items. Each item is scored on a 2-level scale (0, 1, or 2 points). The total score of the OF1-8 scale ranges from 0 to 11, with higher scores indicating more severe oral frailty. A score of >4 is considered high risk for oral frailty.

#### 2.1.6 Sarcopenia

In this cross-sectional study, the SARC-F questionnaire (Strength, Assistance with walking, Rising from a chair, Climbing stairs, and Falls) was employed to assess the risk of sarcopenia among participants. The SARC-F questionnaire was developed by Malmstrom et al. in 2013 and is primarily used for screening sarcopenia in elderly community residents and hospitalized elderly patients (60). It is the most widely used brief tool for screening sarcopenia in the elderly. The SARC-F scale consists of five assessment components: Strength (S), Assistance in walking (A), Rise from a chair (R), Climb stairs (C), and Falls (F). Each component is scored from 0 to 2, and the scores from each component are summed to obtain the final score. The total score of the SARC-F scale ranges from 0 to 10, with a higher score indicating a greater risk of sarcopenia. A SARC-F score greater than 4 suggests the presence of sarcopenia risk (61).

#### 2.1.7 Depressive symptoms

In this cross-sectional study, participants’ depressive symptoms were assessed using the Patient Health Questionnaire-2 (PHQ-2). The PHQ-2 scale is a simplified version of the PHQ-9 and includes two items: “Over the past 2 weeks, have you felt little interest or pleasure in doing things?” and “Have you been feeling down, depressed, or hopeless?” Each item is scored on a 4-point scale from 0 (not at all) to 3 (nearly every day), with a total score ranging from 0 to 6. A higher total score indicates a greater likelihood of depressive symptoms, and a score >3 suggests the presence of depressive symptoms (62).

### 2.2 Data collection

This multicenter cross-sectional survey was conducted via the Wenjuanxing platform,^1^ the most widely used questionnaire software in China. Trained investigators distributed electronic questionnaires to middle-aged and elderly patients with comorbid chronic diseases through one-on-one, face-to-face interactions. After participants signed the electronic informed consent form, they answered the questionnaire by clicking on the electronic link or scanning the questionnaire QR code. If participants had a clear consciousness and good cognitive ability but were unable to complete the questionnaire independently, the investigators conducted one-on-one interviews and then filled out the questionnaire on behalf of the participants based on their responses.

During the investigation process, the investigator provided patient, unbiased answers to all questions raised by the patient throughout the entire procedure. The questionnaire surveyor, who is also the patient’s attending physician and primary nurse, aimed to establish a good level of trust to achieve better data collection outcomes. If participants had difficulty in completing the questionnaire independently, the investigator would use non-leading, neutral language to ask questions about each key point of the scale content and accurately record the patient’s responses. Each questionnaire completed by the participants will undergo rigorous quality checks. The duration of the face-to-face interview was 10–20 min.

### 2.3 Statistical methods

All statistical analyses were performed using SPSS 26.0 for baseline comparisons and R software (version 4.3.0) with the “rms” package for predictive model development and validation. Categorical variables are presented as frequencies (percentages) and were compared between groups using the chi-square test. Correlations among cognitive frailty, oral frailty, sarcopenia, depressive symptoms, and clinical physiological resilience were assessed using Spearman’s rank correlation coefficient, given the categorical nature of these variables.

Study population and data splitting. A total of 752 elderly patients with chronic diseases were enrolled in this study. The total sample was randomly divided into a training set (70%, *n* = 526) and an internal validation set (30%, *n* = 226) using stratified random sampling to ensure balanced outcome distribution. The training set was used for model development, and the validation set was used to evaluate model generalizability.

Missing data. There were no missing data for any of the variables included in this study; therefore, no imputation was required.

Predictor selection and model development. Cognitive frailty was treated as the binary outcome variable. Based on univariate logistic regression analyses (entry criterion: *p* < 0.10) and clinical relevance supported by previous literature, six candidate predictors were considered for model development. To avoid overfitting, the events per variable (EPV) criterion was strictly observed. In the training set (*n* = 526), there were 124 events (cognitive frailty positive cases), yielding an EPV of 20.7, which substantially exceeds the recommended minimum of 10. Multicollinearity among predictors was assessed using the variance inflation factor (VIF), with VIF < 10 indicating no significant multicollinearity. Significant predictors from univariate analysis were then entered into a multivariate binary logistic regression using backward stepwise selection based on the Akaike Information Criterion (AIC) to identify independent risk factors. Model fit was assessed using the Hosmer–Lemeshow goodness-of-fit test, with *p* > 0.05 indicating adequate calibration.

Nomogram construction. A predictive nomogram was constructed based on the independent predictors identified from the final multivariate logistic regression model, providing a visual tool for individualized risk estimation of cognitive frailty.

Model performance evaluation. The performance of the nomogram was comprehensively evaluated in terms of discrimination, calibration, and clinical utility:

Discrimination was assessed using the area under the receiver operating characteristic curve (AUC), with values > 0.80 indicating excellent discriminative ability.

Calibration was evaluated using calibration plots (with bias correction via 1,000 bootstrap resamples) and the Brier score, where lower Brier scores indicate better predictive accuracy.

Clinical utility was assessed using decision curve analysis (DCA) to evaluate the net benefit of the model across a range of threshold probabilities.

Internal validation. To assess model stability and generalizability, the above performance metrics were evaluated in both the training and internal validation sets. Additionally, 1,000 bootstrap resamples were performed within the training set to correct for overfitting optimism and to obtain bias-adjusted calibration curves.

Reporting standards. This study followed the TRIPOD (Transparent Reporting of a Multivariable Prediction Model for Individual Prognosis or Diagnosis) statement for the design, construction, validation, and reporting of the predictive model, ensuring completeness and transparency. All *p*-values were based on two-sided tests, with *p* < 0.05 considered statistically significant.

## 3 Result

### 3.1 Participant characteristics

The characteristics of the participants (*n* = 752) are shown in Table 1. Among the hospitalized older adults with chronic diseases, 55.7% (*n* = 419) were male, and the rest were female, 44.3% (*n* = 333). The age range of the hospitalized elderly patients with chronic diseases was from 60 to 96 years old, with a mean of 71.24 ± 8.136. In this study, the prevalence of cognitive frailty in hospitalized older adults with chronic diseases was 22.74% (171/752). There were significant differences between the depressive symptoms, sarcopenia, oral frailty, gender, age, work status, having a partner, and educational level (*p* < 0.05, Table 1).

**Table 1:** Factors associated with cognitive frailty in univariate analyses.

Characteristics	All participants (*n* = 752)	Cognitive frailty		*χ* ^2^	*p* value
		No (*n* = 581)	Yes (*n* = 171)		
Depressive symptoms				74.185	*p* < 0.001
No	376 (50.0)	340 (90.4)	36 (9.6)		
Yes	376 (50.0)	241 (64.1)	135 (35.9)		
Sarcopenia				170.948	*p* < 0.001
No	455 (60.5)	425 (93.4)	30 (6.6)		
Yes	297 (39.5)	156 (52.5)	141 (47.5)		
Oral frailty				85.634	*p* < 0.001
No	268 (35.6)	258 (96.3)	10 (3.7)		
Yes	484 (64.4)	323 (66.7)	161 (33.3)		
Gender				7.161	*p =* 0.007
Male	419 (55.7)	339 (80.9)	80 (19.1)		
Female	333 (44.3)	242 (72.7)	91 (27.3)		
Age				58.923	*p* < 0.001
60–69	342 (45.5)	303 (88.6)	39 (11.4)		
70–79	286 (38.0)	208 (72.7)	78 (27.3)		
≥80	124 (16.5)	70 (56.5)	54 (43.5)		
Place of residence				5.418	*p =* 0.067
Rural	270 (35.9)	197 (73.0)	73 (27.0)		
Town	156 (20.7)	120 (76.9)	36 (23.1)		
City	326 (43.4)	264 (81.0)	62 (19.0)		
Work status				19.563	*p* < 0.001
At work	128 (17.0)	118 (92.2)	10 (7.8)		
Non-work	624 (83.0)	463 (74.2)	161 (25.8)		
Having a partner				19.339	*p* < 0.001
No	97 (12.9)	58 (59.8)	39 (40.2)		
Yes	655 (87.1)	523 (79.8)	132 (20.2)		
Educational level				35.277	*p* < 0.001
Never attended school	161 (21.4)	105 (65.2)	56 (34.8)		
Primary school	227 (30.2)	167 (73.6)	60 (26.4)		
Junior high school	163 (21.7)	130 (79.8)	33 (20.2)		
High school	102 (13.6)	94 (92.2)	8 (7.8)		
Vocational school	34 (4.5)	26 (76.5)	8 (23.5)		
College degree or above	65 (8.6)	59 (90.8)	6 (9.2)		
Number of surgeries in the past 2 years				1.666	*p =* 0.435
0	272 (36.2)	213 (78.3)	59 (21.7)		
1	277 (36.8)	207 (74.7)	70 (25.3)		
≥2	203 (27.0)	161 (79.3)	42 (20.7)		
Smoking				1.580	*p =* 0.209
No	613 (81.5)	468 (76.3)	145 (23.7)		
Yes	139 (18.5)	113 (81.3)	26 (18.7)		

### 3.2 Analysis of influencing factors on cognitive frailty in elderly patients with chronic diseases

In this study, 171 (22.7%) elderly patients with chronic diseases were identified as having cognitive frailty. Univariate analysis results showed significant differences in depressive symptoms, sarcopenia, oral frailty, gender, age, employment status, partner status, and education level between those with and without cognitive frailty (*p* < 0.01, Table 1).

### 3.3 Correlations between cognitive frailty, oral frailty, sarcopenia, depressive symptoms, and clinical physiological resilience

**Figure 2:**
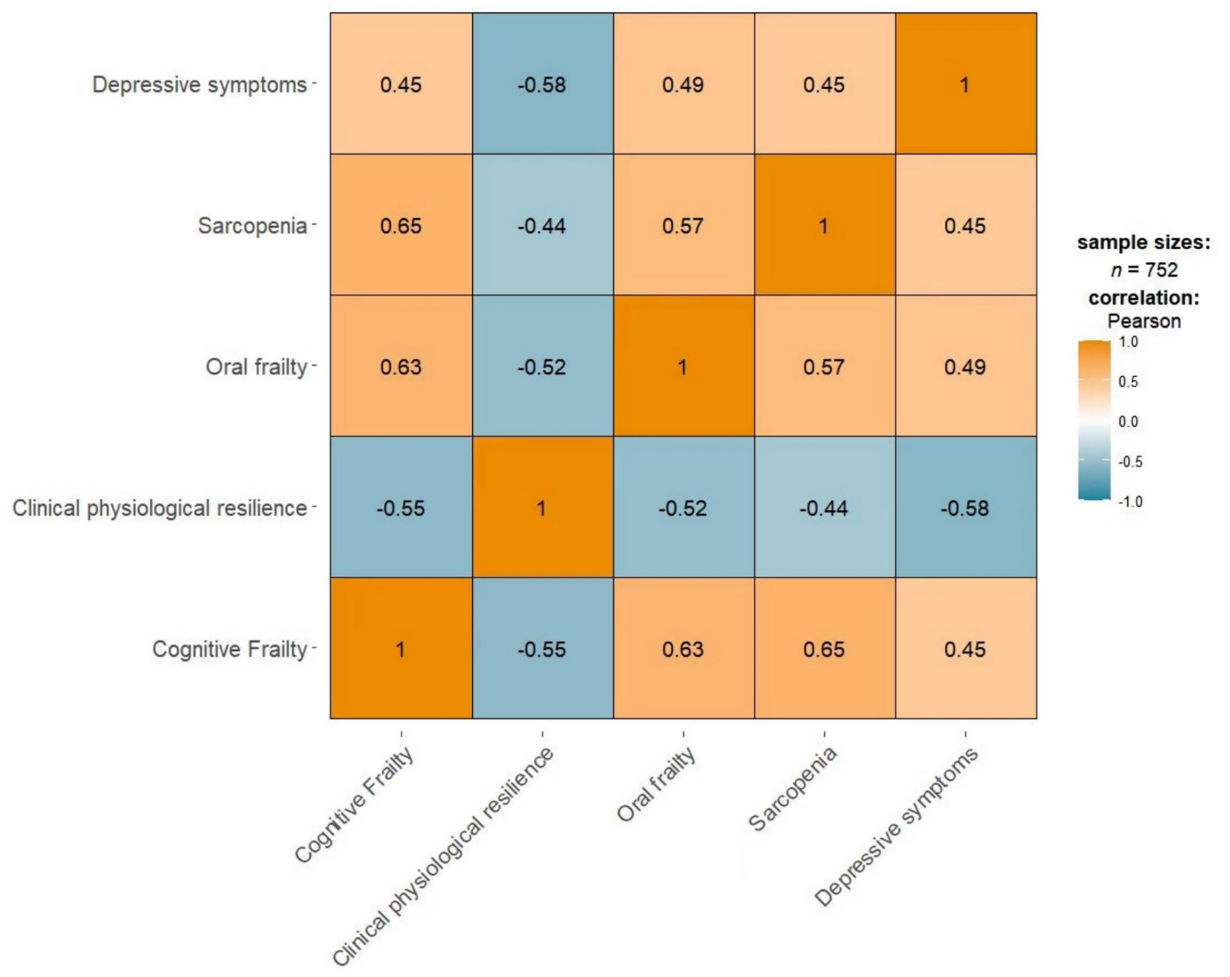
Correlations between cognitive frailty, oral frailty, sarcopenia, depressive symptoms, and clinical physiological resilience.

As shown in Figure 2, there is a significant positive correlation between depressive symptoms, sarcopenia, oral frailty, and cognitive frailty in elderly patients with chronic diseases (*p* < 0.01). In contrast, clinical physiological resilience shows a significant negative correlation with cognitive frailty (*p* < 0.01).

#### 3.3.1 Logistic regression analysis of risk factors for cognitive frailty in elderly patients with chronic diseases

**Figure 3:**
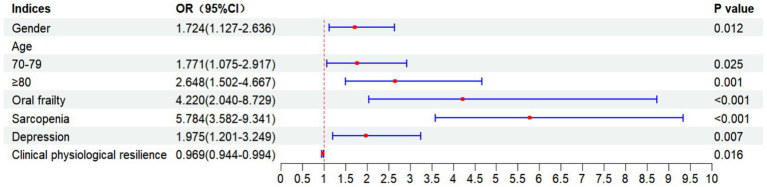
Forest plot of influencing factors for cognitive frailty in elderly patients with chronic diseases.

Chi-square test was used to screen significant variables for inclusion in the binary logistic regression analysis (Table 2). Ultimately, Gender (*p =* 0.012), 70–79 years (*p =* 0.025), ≥80 years (*p =* 0.001), Oral frailty (*p* < 0.001), Sarcopenia (*p* < 0.001), Depressive symptoms (*p =* 0.007), and Clinical physiological resilience (*p =* 0.016) were associated with the development of cognitive frailty in Elderly Patients with Chronic Diseases (Table 2, Figure 3).

**Table 2:** Binary logistic regression analysis of factors associated with cognitive frailty.

Indices	*β*	S.E.	Wald	*p* value	OR	95% CI	Tolerance	VIF
Gender	0.545	0.217	6.319	0.012	1.724	1.127–2.636	0.972	1.029
Age			11.602				0.874	1.144
70–79	0.572	0.255	5.039	0.025	1.771	1.075–2.917		
≥80	0.974	0.289	11.336	0.001	2.648	1.502–4.667		
Oral frailty	1.440	0.371	15.078	<0.001	4.220	2.040–8.729	0.715	1.398
Sarcopenia	1.755	0.245	51.520	<0.001	5.784	3.582–9.341	0.710	1.409
Depressive symptoms	0.681	0.254	7.180	0.007	1.975	1.201–3.249	0.716	1.396
Clinical physiological resilience	−0.032	0.013	5.805	0.016	0.969	0.944–0.994	0.657	1.522
Constant	−3.072	0.830	13.691	<0.001	0.046			

#### 3.3.2 Development of a risk-stratification nomogram based on cross-sectional associations

**Figure 4:**
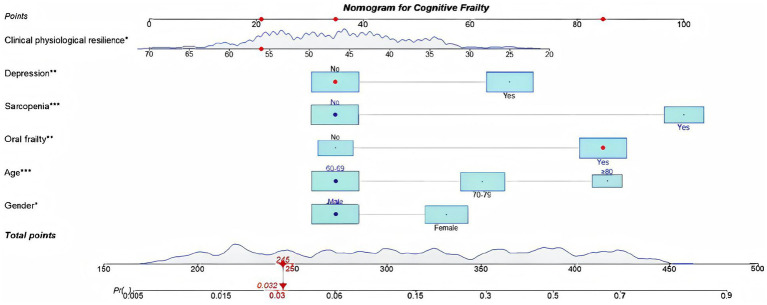
Nomogram for predicting cognitive frailty in elderly patients with chronic diseases.

Given the cross-sectional design of this multicenter study, binary logistic regression was performed to identify factors associated with cognitive frailty and to construct a nomogram for risk stratification, rather than for longitudinal outcome prediction. Variance inflation factor (VIF) tests were conducted, and all variables had VIF values less than 2, indicating no significant multicollinearity. Variables with *p*-values less than 0.05 in the multivariate logistic regression were selected as candidate components for the nomogram. Based on the six independently associated factors (Gender, Age, Oral frailty, Sarcopenia, Depression, and Clinical physiological resilience) identified through the aforementioned univariate and multivariate analyses, the research team utilized the “rms” package in R software to develop a nomogram for estimating the cross-sectional probability of cognitive frailty in this patient cohort (Figure 4). In this nomogram, the position of each variable on its respective axis determines the corresponding score on the point axis, and the total score is calculated by summing the scores of individual factors. For example, for a patient who is male (35 points), has oral frailty (85 points), no depression (35 points), no sarcopenia (35 points), aged 60–69 (35 points), and has a clinical physiological resilience score of 56 (20 points), the total score is 245, corresponding to an estimated probability of cognitive frailty of approximately 0.032. Importantly, given the cross-sectional nature of the underlying data, this nomogram should be interpreted as a screening and risk-awareness tool for identifying high-risk profiles associated with cognitive frailty, rather than as a prognostic instrument for establishing causal relationships or predicting future cognitive decline.

### 3.4 Validation of the risk-assessment model

**Figure 5:**
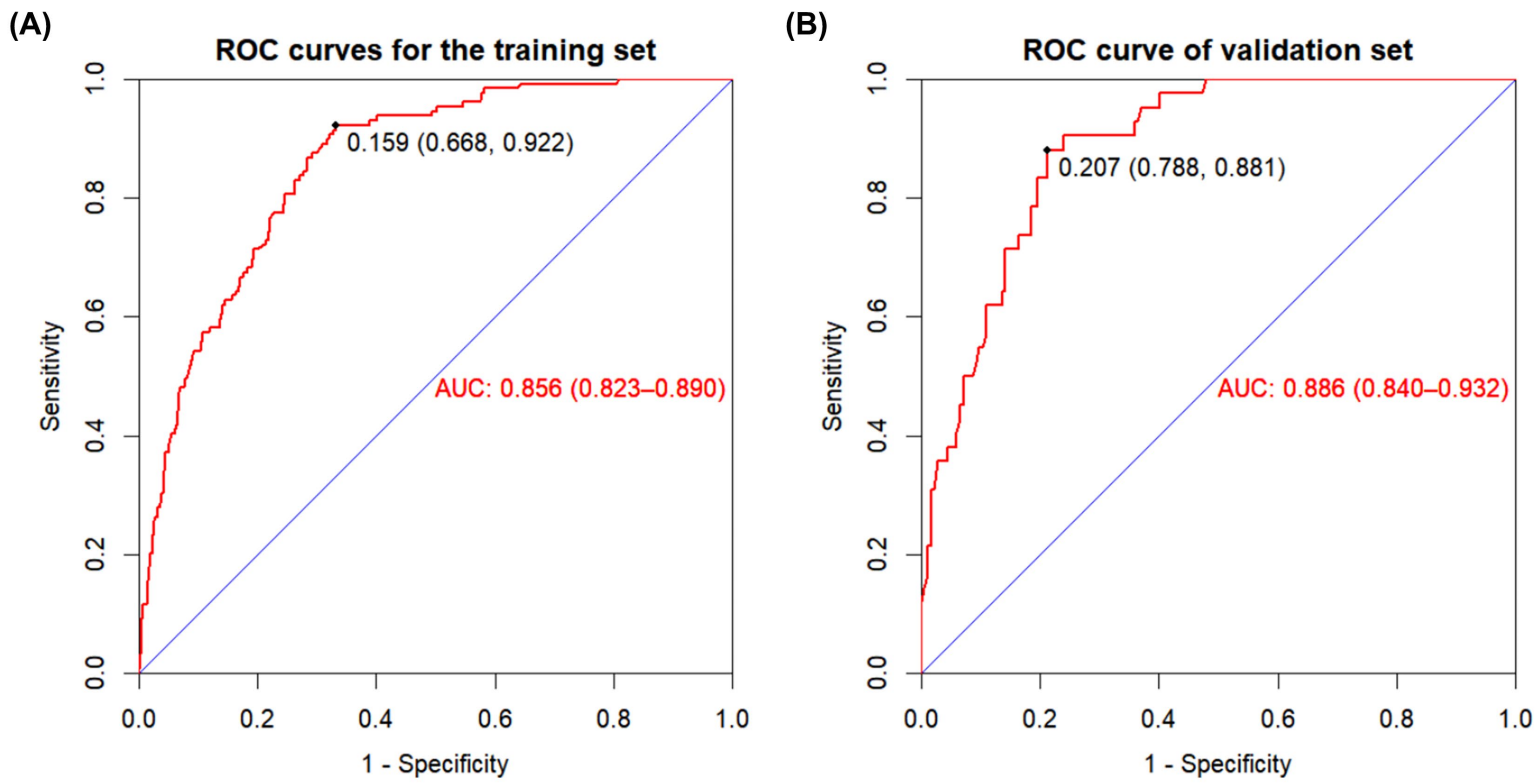
**(A)** Nomogram ROC curve generated from the training dataset. **(B)** Nomogram ROC curve generated from the validation dataset.

The discriminative performance of the predictive model was evaluated by calculating the AUC values of the training and validation datasets. As shown in Figures 5A,B, the model demonstrated excellent performance in both datasets. In the training set, the AUC was 0.856 (95% CI: 0.823–0.890), while in the validation set, the AUC was 0.886 (95% CI: 0.840–0.932). Both the upper and lower limits of the AUC were greater than 0.5, and all values exceeded 0.8, indicating that the model has a very strong discriminative ability and can effectively distinguish between cognitively frail and non-cognitively frail individuals among elderly patients with chronic diseases. This model demonstrates significant clinical value in early screening and intervention, providing crucial support for the effective management of cognitive decline and promoting healthy aging among elderly patients with chronic diseases.

### 3.5 Model calibration and internal validation

**Figure 6:**
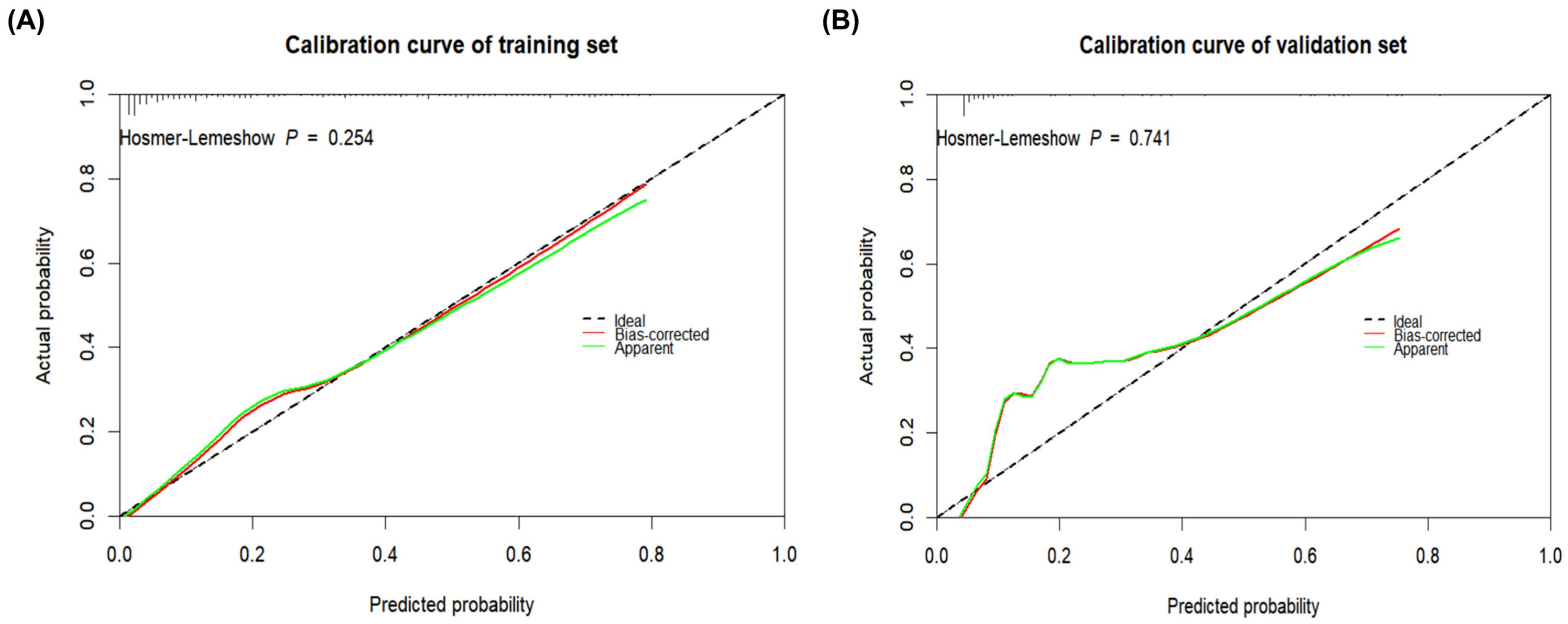
**(A)** Calibration plot for the train dataset. **(B)** Calibration plot for the validation dataset.

The calibration curve and the Hosmer–Lemeshow goodness-of-fit test were used to evaluate the model’s calibration performance (*p* > 0.05 indicates adequate calibration between estimated and observed probabilities). The test results showed that the model exhibited good calibration in both the training set (*χ*^2^ = 10.154, *p* = 0.2054) and the internal validation set (*χ*^2^ = 5.156, *p* = 0.741). The Brier scores for the training set and the internal validation set were 0.128 and 0.104, respectively. The calibration plots for the training set and the internal validation set based on the binary logistic regression model are shown in Figures 6A,B, respectively. In these plots, the black dashed line (ideal) represents the theoretical reference line for perfect agreement between the estimated probabilities and the observed outcomes. The green solid line (apparent) denotes the unadjusted calibration curve derived from the model, while the red solid line (bias-corrected) represents the calibration curve after bootstrap bias correction. Both the calibration curves and the Brier scores indicate a high degree of agreement between the probabilities estimated from the cross-sectional associations and the observed prevalence of cognitive frailty in both the training and internal validation sets. These findings support the nomogram’s potential utility as a risk-awareness and stratification tool for identifying high-risk profiles in this specific cohort, rather than as a prognostic instrument for causal inference or future event prediction.

### 3.6 Clinical validity assessment

The clinical usefulness of the nomogram was evaluated using Decision Curve Analysis (DCA). In both the training and validation sets, the nomogram showed a higher net benefit than the “treat-all” and “treat-none” strategies across a clinically relevant range of threshold probabilities. In the training set, the model provided net benefit when the threshold probability ranged from 0.10 to 0.70, and in the validation set from 0.10 to 0.60.

In clinical practice, these threshold probabilities may represent the level of predicted risk at which clinicians would consider initiating further assessment or intervention. For example, when the predicted probability of cognitive frailty exceeds approximately 10%, patients may be considered for further cognitive screening and frailty assessment. For patients with higher predicted probabilities, more comprehensive management may be warranted, including formal cognitive evaluation, geriatric consultation, nutritional assessment and intervention, oral health assessment, physical function evaluation, and multidisciplinary management when appropriate.

**Figure 7:**
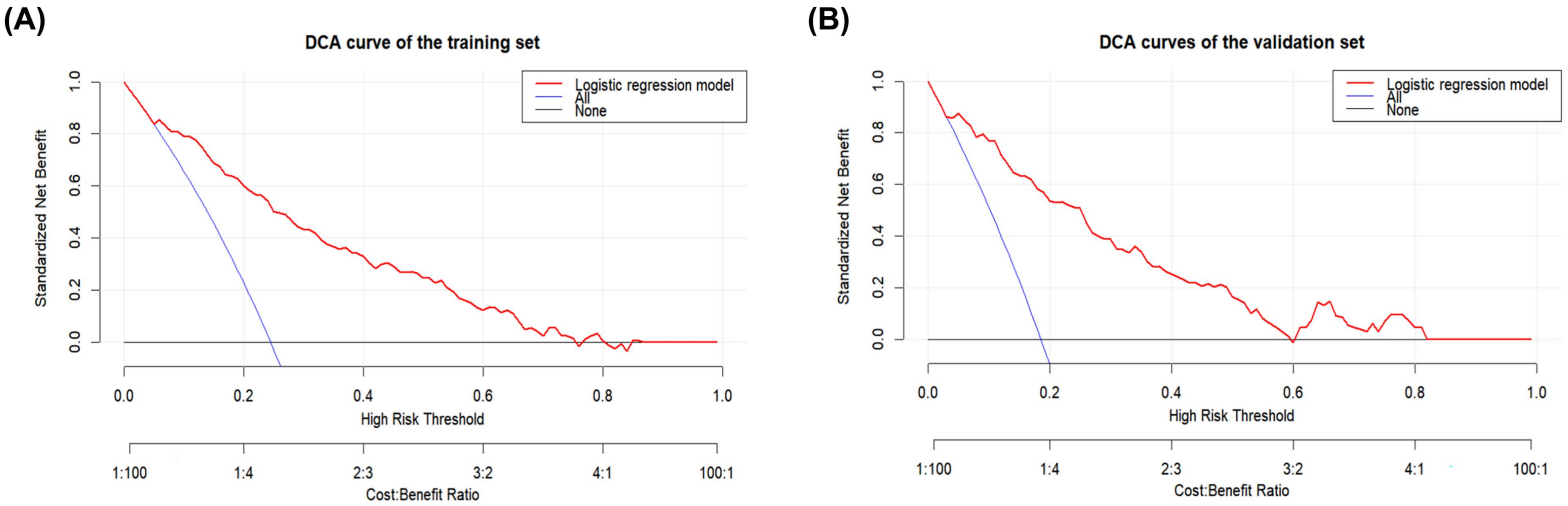
**(A)** DCA curve for the training data set. **(B)** DCA curves for validation data se.

Therefore, the DCA results suggest that the nomogram may help clinicians identify elderly patients with chronic diseases who are at increased risk of cognitive frailty and who may benefit from additional evaluation or early preventive interventions. These findings support the potential clinical applicability and practical value of the model, particularly within the threshold probability ranges shown in Figures 7A,B.

### 3.7 Development of a web-based interactive risk estimator

**Figure 8:**
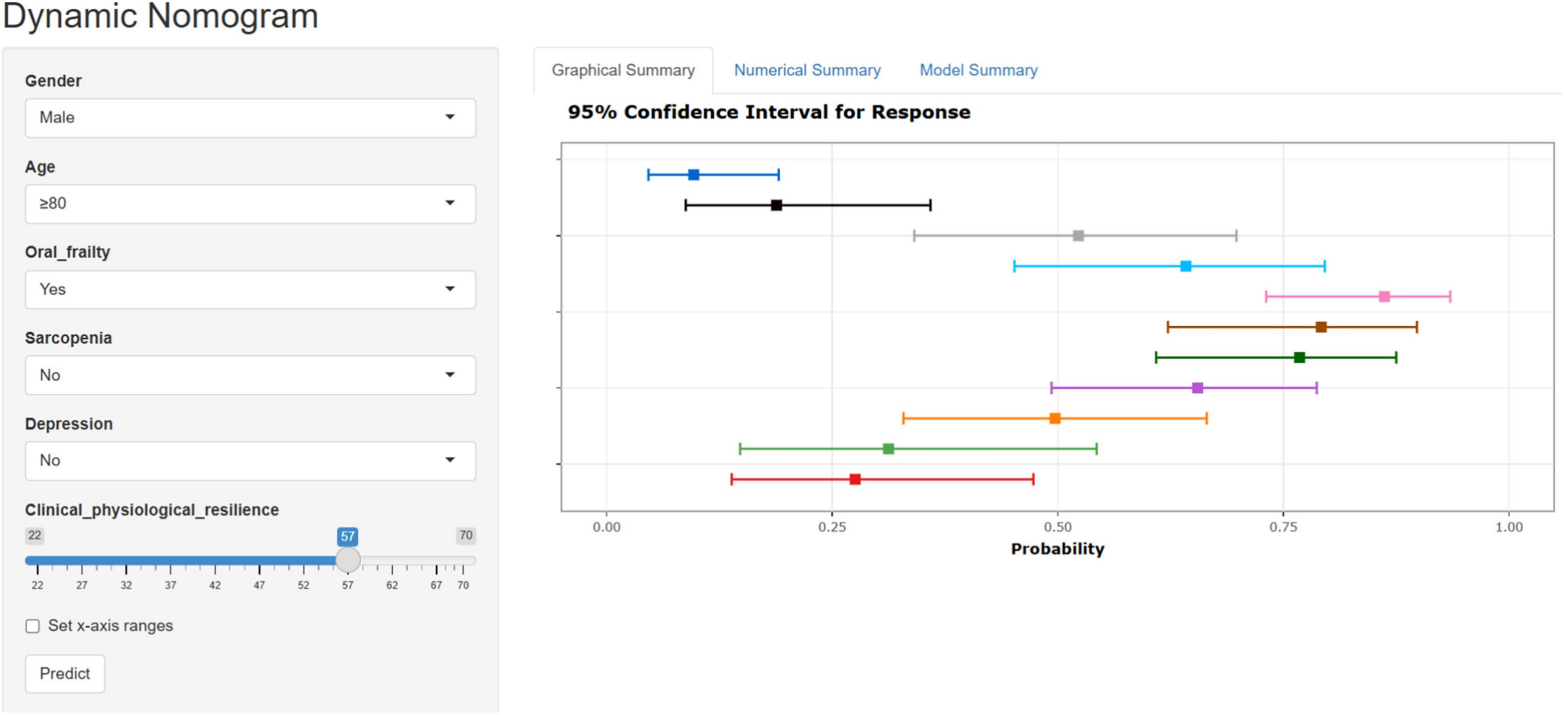
Web-based online nomogram developed through web technologies.

To promote the clinical application of the nomogram, the research team utilized R software and the “DynNom” package to develop a web-based interactive calculator for estimating the probability of cognitive frailty in elderly patients with chronic diseases, based on the cross-sectional associations identified in this study.^2^ By transforming the nomogram into an online risk-awareness and stratification tool, this calculator enables rapid individualized probability estimates, thereby enhancing its convenience for clinical risk screening, patient communication, and targeted intervention planning (Figure 8).

## 4 Discussion

Cognitive frailty is a comprehensive concept that encompasses the manifestations of an individual in both physical frailty and cognitive dysfunction. As individuals age, they are influenced by multiple factors, including socio-demographic characteristics, behaviors and lifestyles, physiological functions and chronic diseases, as well as psychological states and social adaptability. These factors lead to a gradual decline in both physiological and cognitive functions, which interact and influence each other, ultimately resulting in the individual entering a state of cognitive frailty (63). The issue of cognitive frailty in elderly patients with chronic diseases has gradually emerged as a critical challenge in the global public health sector that urgently needs to be addressed. Cognitive Frailty (CF) is a complex syndrome characterized by the co-occurrence of physical frailty and cognitive decline, which not only significantly reduces the patients’ quality of life but also substantially increases the risks of disability, hospitalization, and mortality. In recent years, the pace of population aging in mainland China has accelerated compared to the past. The significant increase in elderly patients with chronic diseases will substantially escalate the societal burden of disease and healthcare demands. Therefore, developing such a screening model is important for identifying cognitive frailty in elderly patients with chronic diseases and for facilitating timely interventions. This model may assist medical staff in identifying hospitalized elderly patients with chronic diseases who are likely to have cognitive frailty and may require further assessment, thereby enabling the timely adoption of appropriate intervention measures.

In this study, the prevalence of cognitive frailty among hospitalized elderly patients with chronic diseases was 22.74%, higher than the incidence rate of cognitive frailty in the elderly population of Qingdao community surveyed by Yang Zhenjing et al. (6.78%) (11), indicating a high level of cognitive frailty in this population (64). The reason may be that the population included in this study consists of elderly inpatients with chronic diseases, whose disease conditions can lead to increased health vulnerability in the elderly. Additionally, the patients experience a decline in physiological reserves, reduced immunity, and diminished stress response capacity due to the impact of their diseases, which increases the incidence of cognitive frailty. Chronic diseases, such as diabetes, stroke, and cardiovascular conditions, have been consistently linked to cognitive decline and frailty, with mechanisms involving systemic inflammation, metabolic dysregulation, and vascular damage (65–67). In summary, the public health issue of cognitive frailty among elderly inpatients with chronic diseases urgently requires attention. This finding not only reveals the health risks specific to this population but also provides a reference basis for clinical interventions and the formulation of public health policies.

This study found a correlation between oral frailty and cognitive frailty. Studies have found that oral health issues can diminish the body’s and mind’s reserve capacities, leading to clinical outcomes such as malnutrition, cognitive impairment, disability, and even death, thereby affecting the quality of life in the elderly (68, 69). Tooth loss is one of the significant indicators of oral frailty, and research has found that tooth loss promotes the occurrence and development of cognitive dysfunction (70). There is a close association between decreased masticatory function and cognitive impairment. Studies have shown that the prevalence of cognitive impairment in elderly individuals with reduced masticatory function is 1.28 times higher than that in elderly individuals with normal masticatory function (71). Furthermore, due to symptoms such as tooth loss, decreased masticatory and swallowing functions, and oral malodor, which affect facial aesthetics and normal communication in the elderly, those with oral frailty are prone to developing feelings of inferiority, leading to reduced daily activities and decreased physical mobility. In elderly individuals, the coexistence of multiple diseases, malnutrition, and inflammatory responses can lead to leukoaraiosis, which affects cognitive function (72). Interventions targeting oral frailty, such as oral rehabilitation training, oral frailty screening, nutritional support, and multidisciplinary collaborative management, may effectively delay the decline of cognitive function. Particularly for elderly chronic disease patients who also suffer from physical frailty, a comprehensive oral frailty health management strategy may be more necessary (73).

Compared to female patients, male patients have a lower prevalence of cognitive frailty, and female was considered a risk factor for cognitive frailty in elderly patients with chronic diseases, which was consistent with the findings of Jie Ren et al. on cognitive frailty in chronic obstructive pulmonary disease (74). From a biological perspective, women are more prone to cognitive frailty in their later years. This may be closely related to the decline in estrogen levels after menopause, as estrogen has a significant protective effect on the nervous system. The reduction in estrogen levels may lead to an accelerated decline in cognitive function (75). And, the role of social and environmental factors in exacerbating cognitive frailty among females cannot be overlooked. Females are more likely to experience social isolation and loneliness, particularly in later life, which has been independently linked to cognitive decline and frailty (76, 77). Moreover, the burden of caregiving responsibilities, which disproportionately falls on females, may contribute to chronic stress and accelerate the progression of cognitive frailty. These factors, combined with the physiological changes associated with menopause, such as reduced estrogen levels, may create a perfect storm for the development of cognitive frailty in females (78). Therefore, from a clinical perspective, the identification of gender as a risk factor for cognitive frailty has important implications for targeted interventions. Preventive strategies should consider sex-specific approaches, such as promoting physical activity and social engagement among females, which have been shown to mitigate the risk of cognitive decline and frailty (77, 79).

This study showed that age is one of the significant independent risk factors for cognitive frailty, which was consistent with the findings (80). The incidence of cognitive frailty varies among elderly individuals of different ages, which may be associated with degenerative changes in the central nervous system, including brain atrophy, and an increased risk of other diseases in the elderly (81). With advancing age, the subclinical pathological states progressively accumulate in the human body, along with age-related complications, which may accelerate the decline in the reserve capacity of multiple organ systems through synergistic effects, thereby inducing systemic homeostatic imbalance and frailty syndrome. This physiological imbalance exerts a significantly selective damaging effect on specific brain regions (such as the medial temporal lobe and the frontostriatal system), manifesting as neuronal dysfunction, reduced synaptic density, and diminished functional reserve capacity, ultimately leading to heterogeneous aging characteristics or cognitive decline in the brain (82).

This study showed that sarcopenia is one of the significant independent risk factors for cognitive frailty, which was consistent with the findings (83). In older adults, sarcopenia is often accompanied by cognitive decline, creating a bidirectional relationship where muscle weakness and reduced physical performance further impair cognitive function, and vice versa (84, 85). This interplay is particularly pronounced in patients with chronic conditions such as cancer, cardiovascular disease, and diabetes, where the catabolic effects of the disease and its treatment accelerate muscle loss and cognitive deterioration (86, 87). The clinical implications of this relationship are profound, as the co-occurrence of sarcopenia and cognitive decline significantly increases the risk of adverse outcomes, including frailty, disability, and mortality (88, 89). Nutritional status plays a critical mediating role in the relationship between sarcopenia and cognitive decline. Malnutrition, a common consequence of chronic diseases, exacerbates both conditions by promoting muscle atrophy and impairing brain function (90, 91). Compared to elderly chronic disease patients without the risk of sarcopenia, those at high risk of sarcopenia had a 2.323 times higher likelihood of developing CF (95% CI = 1.105–4.882). A study conducted by Maria Elena Alvarez Cervantes on 2,158 elderly individuals aged 65 further confirmed that sarcopenia is significantly associated with mild cognitive impairment, and this association persists even after adjusting for other confounding factors (92).

In this study, the finding that depressive symptoms are a risk factor for the development of cognitive frailty in the elderly is consistent with the results of Kwan et al. (93). Kwan et al. (93) suggested that depressive mood can lead to elevated levels of cytokines such as interleukin-6 (IL-6), tumor necrosis factor-alpha (TNF-*α*), and C-reactive protein (CRP). The chronic inflammatory response mediated by these cytokines, on one hand, acts on skeletal muscles, causing a decrease in muscle density and mass, and on the other hand, can cross the blood-cerebrospinal fluid barrier, leading to an increase in amyloid precursor protein in the brain, ultimately resulting in the occurrence of cognitive frailty (94). In recent years, the association between depressive symptoms and cognitive frailty in elderly patients with chronic diseases has become a significant topic in the fields of geriatric medicine and neuroscience. Numerous studies have shown that depressive symptoms are not only common comorbidities among elderly patients with chronic diseases but also one of the key risk factors leading to cognitive frailty (95). In the elderly population with cognitive frailty, group-based interventions such as targeted health education and psychological sharing are commonly implemented (96–98), encouraging increased social participation to enhance their self-respect, self-achievement, self-worth, and self-efficacy.

This study found that clinical physiological resilience is a protective factor against frailty in hospitalized older adults with chronic diseases. Our study builds upon existing literature by elucidating the interplay between physiological resilience and cognitive frailty in a vulnerable population of elderly inpatients with chronic diseases. The results suggest that diminished physiological resilience exacerbates the risk of cognitive frailty, highlighting the need for targeted interventions to enhance resilience and mitigate this dual burden (99). The association between physiological resilience and cognitive frailty can be partially explained by the cumulative impact of chronic diseases on both physical and cognitive health. Chronic diseases, such as diabetes, stroke, and cardiovascular conditions, are well-documented risk factors for cognitive decline and frailty, often through mechanisms involving systemic inflammation, oxidative stress, and metabolic dysregulation. In our study, participants with lower physiological resilience exhibited a higher prevalence of these chronic conditions, which likely contributed to their increased susceptibility to cognitive frailty. This aligns with prior research indicating that the management of chronic diseases is crucial for preserving cognitive function and physical robustness in older adults. Furthermore, the interplay between physiological resilience and cognitive frailty may be mediated by factors such as nutritional status, sleep quality, and mental health, which are known to influence both cognitive and physical resilience.

Cognitive frailty among elderly patients with chronic diseases has emerged as an increasingly significant public health issue, especially against the backdrop of the rapidly aging global population. This condition not only markedly diminishes the quality of life for elderly individuals suffering from chronic illnesses but also substantially elevates the strain on the healthcare system. Intervention strategies for cognitive frailty require multidisciplinary collaboration and personalized design. This cross-sectional study reveals that cognitive frailty is closely associated with multiple factors, encompassing clinical physiological resilience, depressive symptoms, oral frailty, and sarcopenia. In light of this, interventions for cognitive frailty should comprehensively cover various dimensions, including nutritional support, psychological counseling, and social activity participation. Healthcare institutions and policymakers should actively advocate for interdisciplinary team collaboration, meticulously craft targeted comprehensive intervention plans, and leverage health promotion programs to widely disseminate such plans to a larger population of elderly patients with chronic diseases. In summary, identifying and implementing targeted interventions for cognitive frailty in the early stages can help optimize the allocation of medical resources and alleviate the burden associated with cognitive frailty. Clarifying the risk factors of cognitive frailty enables the provision of personalized care measures for elderly patients with chronic diseases, allowing for more precise prevention or improvement of their cognitive frailty status, thereby enhancing their quality of life and promoting healthy aging.

### 4.1 Limitations

Although our cross-sectional study has provided valuable insights, there are still some inevitable limitations that need to be addressed. Firstly, as cross-sectional studies do not involve causal inference, further longitudinal research is needed to explore the potential mechanisms. Secondly, the self-reported information from participants in this study may be subject to recall bias; for instance, the PHQ-9 scores and AD-8 scale, serving as self-reported screening tools rather than clinical diagnostic instruments, may yield results that deviate from the actual situation. Thirdly, the prevalence estimates of cognitive frailty obtained through convenience sampling are significantly influenced by sampling bias. In future studies, we plan to adopt more rigorous and reasonable sampling methods to enhance the robustness and generalizability of the research findings.

## 5 Conclusion

This study shows that the prevalence of cognitive frailty is high among hospitalized older adults with chronic diseases and is associated with gender, age, oral frailty, sarcopenia, and depressive symptoms, while clinical physiological resilience appears to be a protective factor in terms of concurrent association. Based on this cross-sectional multicenter dataset, we successfully developed and internally validated a nomogram for estimating the probability of cognitive frailty in this population. Our nomogram integrates factors including clinical physiological resilience, depression, and oral frailty, and has undergone internal validation, proving to be a useful risk-stratification and screening tool. This association-based tool may assist clinicians in identifying older adults with chronic diseases who have a higher probability of concurrent cognitive frailty, thereby facilitating early risk awareness and targeted comprehensive geriatric assessments. Moreover, our findings have important implications for developing screening and stratification strategies in clinical practice. From a public health perspective, this study provides a valuable reference for identifying high-risk profiles and guiding preventive interventions for cognitive frailty.

## Data Availability

The raw data supporting the conclusions of this article will be made available by the authors, without undue reservation.

## Footnotes

1
https://www.wjx.cn/

2
https://lqwlqw.shinyapps.io/Cognitive_frailty_Web/
